# Experimental Investigation of a C-S-H Nanocrystalline Nucleus Modified with PCE Dispersant on the Early-Age Mechanical Behavior of Oil Well Cement Paste

**DOI:** 10.3390/ma18020326

**Published:** 2025-01-13

**Authors:** Xiujian Xia, Yongjin Yu, Fengzhong Qi, Pengpeng Li, Congfeng Qu, Pu Xu, Huiting Liu, Renzhou Meng, Xianzhi Zhai, Jintang Guo

**Affiliations:** 1 CNPC Engineering Technology R&D Company Limited, No. 5 Huanghe Street, Changping, Beijing 102206, China; xiaxiujiandr@cnpc.com.cn (X.X.); yuyongjindri@cnpc.com.cn (Y.Y.); qfz69dri@cnpc.com.cn (F.Q.); qucongfengdri@cnpc.com.cn (C.Q.); xupdr@cnpc.com.cn (P.X.); liuhtdr@cnpc.com.cn (H.L.); mengzrdr@cnpc.com.cn (R.M.); zhaixianzhidr@cnpc.com.cn (X.Z.); 2 National Engineering Research Center of Oil & Gas Drilling and Completion Technology, No. 5 Huanghe Street, Changping, Beijing 102206, China; 3 School of Chemical Engineering and Technology, Tianjin University, No. 135 Yaguan Road, Jinnan, Tianjin 300354, China; jtguo@tju.edu.cn

**Keywords:** nanocrystalline nucleus, early strength, early hydration, hydrated calcium silicate, OWC paste

## Abstract

For the exploration and development of oil and gas reservoirs in shallow, cold regions and deep oceans, oil well cement (OWC) pastes face the challenge of slow cement hydration reactions and the low early-strength development of cement stone at low temperatures, which can cause the risk of fluid channeling and the defective isolation of the sealing section during the cementing construction process. To address the above challenges, a nanoscale hydrated calcium silicate (C-S-H) crystal nucleus, DRA-1L, was synthesized. Its application performance and action mechanism were studied. The structural characterization of DRA-1L revealed that its crystal structure resembles that of amorphous C-S-H gel, with a size distribution ranging from 20 to 200 nm. The addition of DRA-1L significantly shortens the transition time of static gel strength, preventing the channeling of OWC paste and promoting the strength development of cement stone at low temperatures. Moreover, the mechanism by which DRA-1L enhances the early strength of cement stone was studied. Results indicated that the nanoscale DRA-1L with nucleation effect reduces the barrier to C-S-H gel formation and accelerates cement hydration, which leads to the increased compactness and early strength of cement stone.

## 1. Introduction

As the exploration and development of oil/gas resources continue to expand, cementing operations have become increasingly crucial, particularly in shallow, extreme cold regions and deep oceans. However, low temperatures can slow down the hydration reaction and prolong the setting time of OWC paste, which leads to annular gas migration during the cementing operation and defective isolation of the sealing section [1,2]. Ensuring cementing quality, reducing well construction time, and lowering the costs of oil and gas exploration and development become challenging when cement has a long setting time and low early strength [3]. Early strength agents, also known as accelerators, are typically used to shorten the setting time and enhance the early strength of OWC paste [4]. Currently, common early strength agents for oil well cement (OWC) paste include calcium chloride, sulfate (such as sodium sulfate) and organic amines (e.g., triethanolamine) [5,6,7]. While calcium chloride is an effective early strength agent, it can negatively affect the thickening of OWC paste, and chloride ions are corrosive to well casings and other metal materials. Sulfates are not conducive to early-strength development at low temperatures. Organic amines are overly sensitive and can easily cause the super retardation of OWC paste. Consequently, the application of these commonly used early strength agents is limited in low-temperature cementing engineering.

With the continuous and intensive study of the hydration mechanism of oil well cement (OWC), the synthesis of nano hydration products has become the focus in the petroleum and construction industries [8,9,10]. Calcium silicate hydrate gel (C-S-H) is the primary hydration product of Portland cement and is crucial for the strength growth of inorganic cementing materials, such as OWC. It plays a significant role in cement hydration and greatly contributes to the mechanical strength development of cement stone. Reports indicate that the induction of crystal nuclei and in situ growth of C-S-H can accelerate cement hydration reactions and improve the compactness and mechanical strength development of cement stone, thereby avoiding the erosion and damage of the cement stone structure caused by introduced harmful ions [11,12,13,14]. Currently, various methods are available for synthesizing C-S-H, including the hydrothermal method, single-ore hydration method, and chemical precipitation method [15,16,17]. In this study, calcium nitrate and sodium silicate were used as calcium and silicon sources, respectively. A solution chemical precipitation method at low temperature was employed to prepare the C-S-H nanocrystalline nucleus, which is the early strength agent for OWC paste. Its comprehensive performance has been thoroughly studied.

## 2. Materials and Methods

### 2.1. Materials

Sodium silicate nonahydrate (Na_2_SiO_3_·9H_2_O, AR) and calcium nitrate tetrahydrate (Ca(NO_3_)_2_·4H_2_O, AR) were obtained from Shanghai Aladdin Co., Ltd. (Shanghai, China). Polycarboxylic acid dispersant (PCE, solid content of 40 wt%) was purchased from Shenyang Benxi Wanhate Chemical Co., Ltd. (Shenyang, China). The Class G OWC (OWC, G grade, high sulfate resistant) used in this study was provided by Sichuan Jiahua Special Cement Co., Ltd. (Leshan, China). The mineral and chemical compositions of OWC, listed in Table 1, were determined by X-ray diffraction (XRD) with Rietveld analysis and X-ray fluorescence (XRF), respectively. The additives used in the OWC paste were obtained from Hebei Zhuozhou Jingxi Petroleum Engineering Technology Co., Ltd. (Zhuozhou, China).

### 2.2. Design of Nanocrystalline Nucleus for Use as Early Strengthening Agent

The C-S-H gel in the hydration products is the primary factor responsible for the solidification of the OWC paste and the early-strength development of cement stone during the cement hydration process. Therefore, introducing synthesized C-S-H crystal nuclei into the OWC paste to provide nucleation sites for the generation and growth of hydration products is expected to promote cement hydration and accelerate the early-strength development of cement stone. The design ideas mainly include: (1) C-S-H crystal nuclei are prepared by a low-temperature chemical precipitation method in the presence of PCE. (2) The nanocrystalline nucleus of C-S-H provides a template to reduce the C-S-H generation barrier, induce the in situ growth of hydration products on the nucleus, and accelerate the formation of C-S-H gel. (3) The rapid dissolution of C_3_S and the formation of calcium hydroxide (CH) crystals accelerate the cement hydration process, shortening the induction period. (4) The semi-crystalline or amorphous phase, with a large specific surface area, disperses the growth of hydration products, thereby densifying the cement stone. (5) The PCE used to modify the surface of the nanocrystalline nucleus can disperse and stabilize the system, avoiding negative effects on the hydration and rheology of the OWC paste.

### 2.3. Preparation and Characterization of Nanocrystalline Nucleus

**Figure 1:**
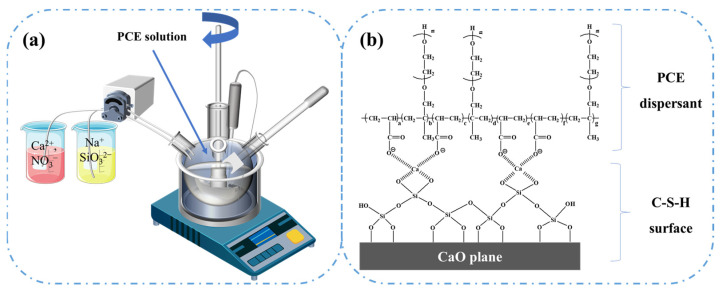
Preparation process (**a**) and molecular structure schematic diagram (**b**) of the nanocrystalline nucleus.

First, sodium silicate solution and calcium nitrate solution, each with a concentration of 30%, were prepared. Then, the sodium silicate solution and calcium nitrate solution were separately added dropwise into the PCE solution using a peristaltic pump with high-speed stirring at 30 °C. After 3 h of reaction, a stable milky white suspension of C-S-H crystal nuclei was obtained, with a concentration of 25%. A schematic diagram of the molecular structure of the nanocrystalline nuclei is shown in Figure 1. A series of C-S-H crystal nuclei with different Ca/Si ratios was obtained by adjusting the concentration ratio of Na_2_SiO_3_ and Ca(NO_3_)_2_. The purified nanocrystalline nuclei were collected by centrifugation and washed several times with water.

The chemical compositions and structures of the pure nanocrystalline nuclei were analyzed using a Nicolet 6700 Fourier infrared spectrometer (Thermo Nicolet Corporation, Madison, WI, USA) through the KBr pellet method. The measurements were taken over a spectral range of 400 to 4000 cm^−1^ with 32 scans [18]. The crystal structures were examined via X-ray diffraction (D8 Advance, Bruker, Berlin, Germany). Samples were scanned from 5° to 70° at a rate of 7°/min [19]. Differences in the composition of the nanocrystalline nuclei were assessed using X-ray fluorescence (XRF-1800, Shimadzu, Kyoto, Japan). The thermal stability of the nanocrystalline nuclei was investigated using a thermal gravimetric analyzer (TGA-Q500, Shimadzu, Japan) in a nitrogen atmosphere. The heating rate was set at 10 °C per min, with a temperature range from 25 °C to 600 °C [20]. The size distribution of nanocrystalline nuclei was measured using dynamic light scattering method with particle size analyzer (Malvern, Worcestershire, UK). Additionally, the microscopic morphology of the nanocrystalline nuclei was characterized using Scanning Electron Microscopy (JEOL, Kyoto, Japan) and Transmission Electron Microscopy (JEOL, Japan).

### 2.4. Preparation and Evaluation of OWC Paste

The OWC pastes were prepared in accordance with the Recommended Practice for Testing Well Cements outlined in API Recommended Practice 10B-2 [21]. The basic OWC paste consisted of OWC and a specific dosage of DRA-1L (0/0.75/1.5/3.0/4.5 wt% by weight of cement, bwoc) and 44 wt% bwoc water. The fundamental properties of the OWC paste were subsequently measured.

The influence of the nanocrystalline nucleus on the rheological properties of the OWC paste was assessed using a viscometer (ZNN-D6, Rongjida, Shanghai, China). The fluidity was evaluated by measuring the expanded diameter of OWC pastes in a frustoconical mold on a specialized jumping table. The thickening time (TT) of the OWC paste was determined at various temperatures [21]. The impact of the nanocrystalline nucleus on compressive strength was investigated using an OFITE compressive strength tester. The specimens were obtained by curing the OWC paste in molds (5.08 cm × 5.08 cm × 5.08 cm) at a specific temperature. When the test temperature was below 93 °C, the OWC pastes were cured at atmospheric pressure using a TG-1280A curing box. If the temperature exceeded 93 °C, the OWC pastes were cured in a chamber with 21 MPa using a TG-7073D dual cylinder HTHP maintenance chamber. For both early- and post-stage compressive strength values, the average of three independent samples was calculated. A static gel strength analyzer (5265, Chandler Engineering, Tulsa, OK, USA) was employed to record the compressive strength and static gel strength development process of cement stone in real time using the ultrasonic method [18].

The effect of the nanocrystalline nucleus on the hydration process of the OWC paste was characterized using XRD, TG-DTG, and cement hydration heat measurements [22].

## 3. Results and Discussion

### 3.1. Optimization of the Ca/Si Molar Ratio of Nanocrystalline Nucleus

The C-S-H nanocrystalline nucleus for the OWC paste was prepared using a solution chemical precipitation method in the presence of PCE at low temperature. The effect of the Ca/Si ratio of the nanocrystalline nucleus on the early compressive strength development of the OWC paste was investigated, and the results are presented in Table 2. As the Ca/Si molar ratio increased, the early compressive strength of the OWC paste initially increased and then decreased. When the Ca/Si molar ratio was 1.75, the compressive strength of the OWC paste reached its peak, achieving 18.0 MPa and 29.1 MPa after curing at 60 °C for 4 h and 8 h, respectively. Additionally, compared to the OWC paste without the nanocrystalline nucleus (No. 1#), the addition of the nanocrystalline nucleus with a Ca/Si molar ratio of 1.75 (No. 5#) increased the compressive strength of the OWC paste by 200% and 83.6% after curing for 4 h and 8 h, respectively. Consequently, a Ca/Si molar ratio of 1.75 was chosen to prepare the nanocrystalline nucleus for use as an early strength agent (designated as DRA-1L) in low-temperature OWC paste.

### 3.2. Structural Characterization of DRA-1L

#### 3.2.1. X-Ray Diffraction (XRD) Analysis

**Figure 2:**
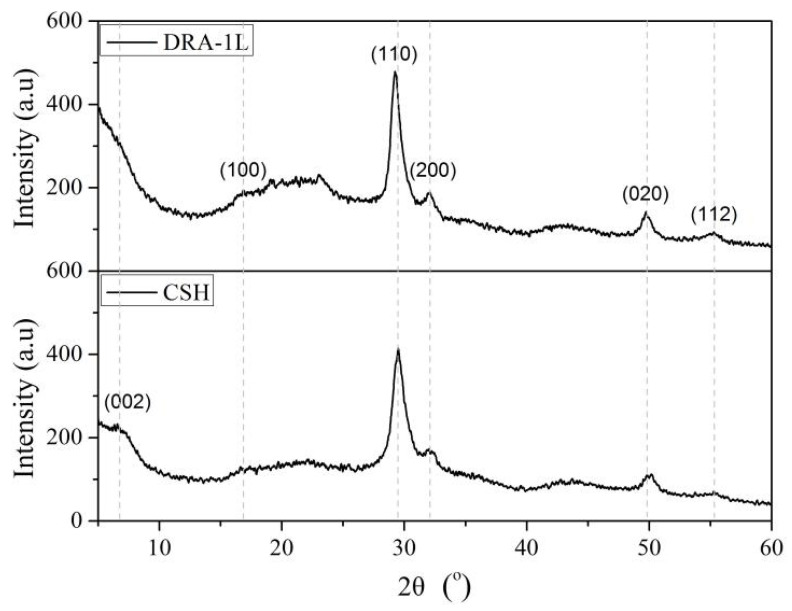
XRD patterns of C-S-H and DRA-1L.

The XRD patterns of the purified C-S-H and DRA-1L samples are shown in Figure 2. It can be observed that C-S-H exhibits distinct characteristic diffraction peaks at 7° (d_002_), 29.5° (d_110_), 32.1° (d_200_), 49.9° (d_020_), and 55.4° (d_112_) of 2θ, indicating its semi-crystalline nature. However, the characteristic diffraction peak of DRA-1L at 7° of 2θ has disappeared, suggesting that the PCE in the system induces distortion of the C-S-H crystal structure, leading to a decrease in crystallinity. Additionally, compared to C-S-H, the intensity of the characteristic diffraction peaks for the C-S-H phase in DRA-1L at 29.5°, 32.1°, 49.9°, and 55.4° of 2θ has increased. This is attributed to the adsorption of PCE, which has a long side-chain structure, on the surface of the C-S-H semi-crystalline phase structure, thereby increasing the interlayer spacing. These results align with the related literature [23]. Consequently, the early strength agent DRA-1L is characterized as a gel solution of semi-crystalline C-S-H.

#### 3.2.2. FT-IR Analysis

**Figure 3:**
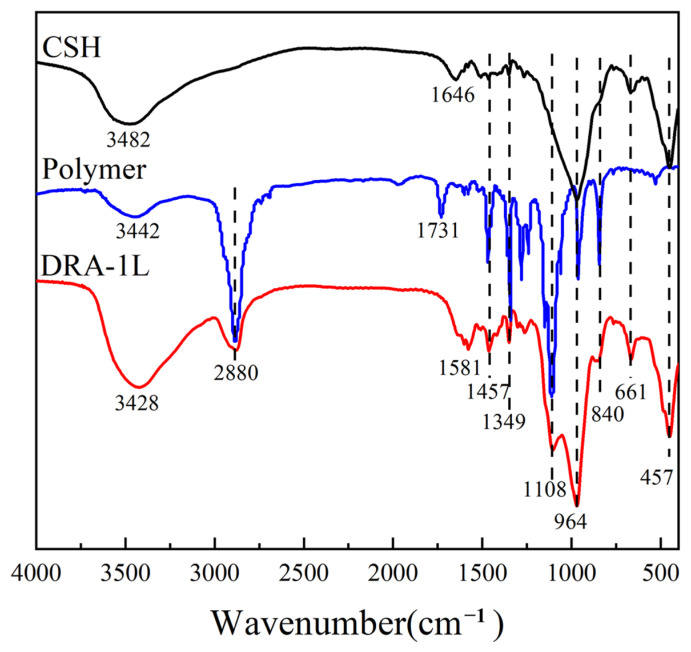
FT-IR spectra of C-S-H, polycarboxylic acid dispersant, and DRA-1L.

The chemical structures of C-S-H, polycarboxylic acid dispersant, and DRA-1L were analyzed using FT-IR measurements, and the results are shown in Figure 3. The symmetric stretching vibration peak of Si-O-Si in C-S-H is located at 661 cm^−1^, the stretching vibration peak of Si-O is at 964 cm^−1^, and the characteristic absorption peak of O-H is situated at 3428 cm^−1^. The peak at 1646 cm^−1^ can be attributed to the flexural vibration of O-H from water chemically adsorbed on the crystalline structure of KBr. The stretching vibration peaks of -CH and -CH_3_ in the PCE dispersant are located at 1261 cm^−1^ and 2880 cm^−1^, respectively. The characteristic absorption peak of -COO^−^ in the PCE dispersant appears at 1731 cm^−1^. All the characteristic absorption peaks of C-S-H and PCE are presented in the infrared spectrum of DRA-1L, indicating that DRA-1L is a product of C-S-H modified by PCE.

#### 3.2.3. X-Ray Fluorescence (XRF) Analysis

The chemical components of the early strength agent DRA-1L were characterized using an XRF spectrometer and the results are presented in Table 3. DRA-1L is primarily composed of Ca and Si, and its chemical composition closely resembles that of C-S-H with a C_3_S_2_H_3_ structure (3CaO·2SiO_2_·3H_2_O) [24]. The results confirm the successful synthesis of C-S-H.

#### 3.2.4. Thermal Stability Analysis

**Figure 4:**
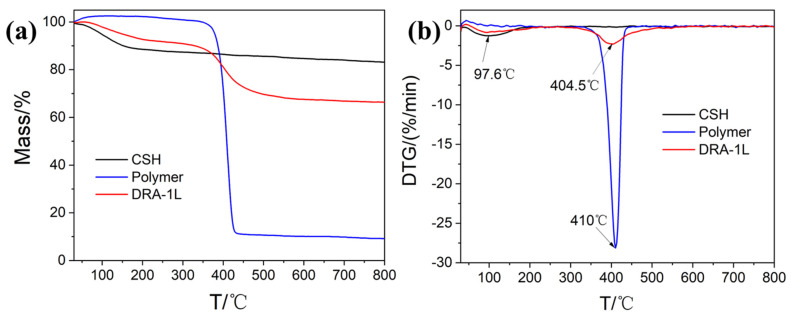
TG (**a**) and DTG (**b**) curves of C-S-H, PCE, and DRA-1L.

The thermogravimetric (TG-DTG) analysis was employed to investigate the thermal behavior of the synthesized DRA-1L. The TG-DTG curves for C-S-H, PCE, and DRA-1L are displayed in Figure 4. For the C-S-H and DRA-1L samples, the exothermic peaks were observed at 97.6 °C and 410 °C, respectively. In the case of C-S-H, a mass loss of 16.6% occurred between 30 °C and 350 °C due to the dehydration of crystal water, indicating a structure similar to that of C_3_S_2_H_3_. For the PCE sample, rapid mass breakdown began above 368 °C, with a maximum rate of about 410 °C, which proves that PCE dispersants have excellent temperature resistance and stable molecular structure. For the sample DRA-1L, two exothermic peaks appeared on the thermogravimetric curve, primarily resulting from the dehydration of the crystal water and the chain scission of the grafted polymer. This indicates that the PCE dispersant had been successfully grafted onto the surface of the C-S-H sample.

The thermogravimetric analysis can also qualitatively elucidate the interaction between the PCE dispersant and the synthetic C-S-H. The TG-DTG curves of DRA-1L reveal that the mass loss in the temperature range of 30~200 °C is primarily attributed to the volatilization of the adsorbed water and interlayer water in the sample. In contrast, the mass loss in the temperature range of 220~550 °C is mainly due to the oxidation and breakage of the molecular chain of the PCE dispersant. Essentially no mass change occurs in the temperature range of 550~800 °C. Notably, the temperature range of the maximum exothermic peak for the PCE dispersant in DRA-1L is broader than that of the PCE dispersant alone. These analyses suggest that the PCE in DRA-1L is present in the C-S-H structure in two ways: surface grafting and interlayer insertion. The mass loss at lower temperatures is caused by the oxidation and rupture of the PCE main chain grafted or adsorbed on the C-S-H surface. Meanwhile, the mass loss at higher temperatures results from the oxidation and rupture of the PCE between the layers of C-S-H.

#### 3.2.5. Microscopic Morphology of DRA-1L

**Figure 5:**
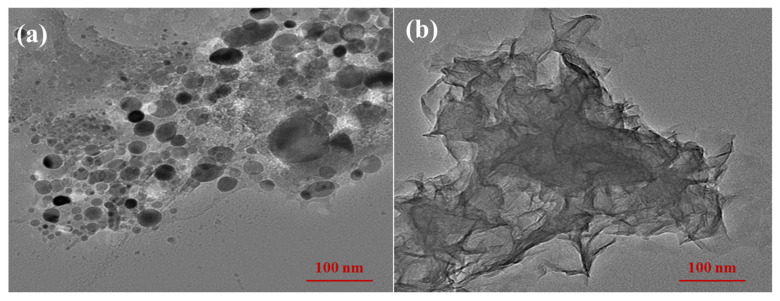
Microscopic morphology of DRA-1L: (**a**) SEM, (**b**) TEM.

The microscopic morphology of DRA-1L was characterized using SEM and TEM measurements. As shown in Figure 5a, the suspension solution of DRA-1L contains numerous spherical particles and aggregates, which are the products of the non-classical nucleation effect during the dropwise addition process using the chemical precipitation method. Additionally, the sample features many foil-like products with nanometer thickness (Figure 5b), providing ample sites for the grafting and interlayer adsorption of the polycarboxylic acid dispersant on the C-S-H surface. Consequently, DRA-1L possesses a semi-crystalline phase structure with a large specific surface area, which facilitates the dispersed growth of cement hydration products and enhances the mechanical properties of cement stone through nucleation and filling effects.

#### 3.2.6. Size Distribution of DRA-1L

**Figure 6:**
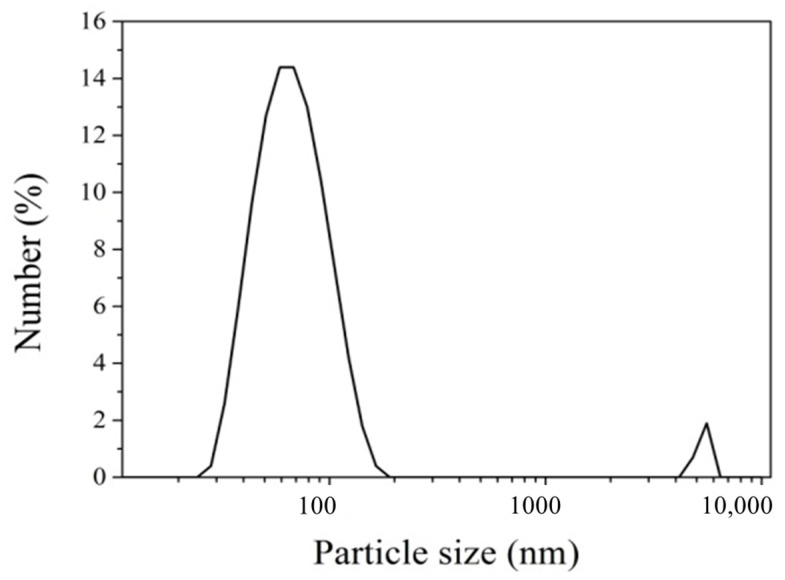
Size distribution of DRA-1L.

The particle size and particle size distribution of DRA-1L were characterized using dynamic light scattering (DLS). The particle size distribution of DRA-1L exhibits a bimodal distribution, ranging from 20 to 200 nm with an average particle size of 90 nm, indicating that it is a crystalline nucleus product at the nanoscale (Figure 6). The synthesized C-S-H particles tend to aggregate and settle due to surface charge. However, the adsorption of PCE dispersant on their surfaces causes the aggregated particles to dissociate into smaller ones. The surface characteristics of C-S-H are altered by the steric hindrance and surface lubrication of the long side chains of polymers among particles, preventing them from aggregating and settling, and thereby maintaining the stability of the system [25].

### 3.3. Properties Evaluation of DRA-1L on OWC Paste

#### 3.3.1. Rheological Properties of OWC Paste

The rheology of OWC paste is the fundamental parameter in rheology design, playing a crucial role in preventing formation leakage, ensuring construction safety, and enhancing displacement efficiency. Traditional inorganic salt early strength agents or coagulants usually have negative effects on the consistency and fluidity of OWC paste, even when used in small amounts. Therefore, the effect of DRA-1L on the rheological properties of OWC paste was investigated. Results are presented in Table 4. As shown in Table 4, the OWC paste without DRA-1L (0% bwoc DRA-1L) exhibited an initial large shear force, strong thixotropy, and high consistency. Compared to the OWC paste without DRA-1L, the fluidity index (*n*) value of the OWC paste with DRA-1L increased, while the consistency coefficient (*K*) value decreased. It can be observed that the fluidity of OWC paste is improved by the addition of DRA-1L, and the fluidity remains almost unchanged with the increasing dosage of DRA-1L. The nanosize effect of C-S-H nanocrystals and the adsorption and dispersion of PCE dispersants are in equilibrium, allowing DRA-1L to significantly enhance the rheological properties of OWC paste, with minimal influence from the dosage of DRA-1L [26]. DRA-1L can improve the mechanical properties of cement stone by increasing its dosage while meeting the rheological requirements of OWC paste.

#### 3.3.2. Thickening Performance of OWC Paste

**Figure 7:**
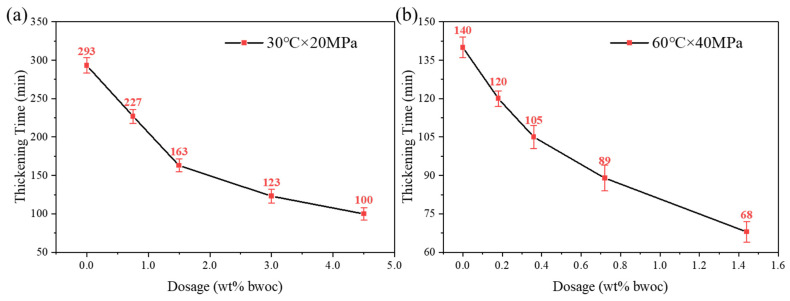
The effect of DRA-1L on the thickening time of OWC paste: (**a**) 30 °C × 20 MPa, (**b**) 60 °C × 40 MPa.

The effect of DRA-1L dosage on the thickening performance of OWC paste was investigated at 30 °C (0/0.75/1.50/3.0/4.5 wt% bwoc) and 60 °C (0/0.18/0.36/0.72/1.44 wt% bwoc). As depicted in Figure 7, the addition of DRA-1L significantly shortens the thickening time of OWC paste at low temperatures. The relationship between thickening time and DRA-1L dosage is linear at low dosages, but the rate of decrease slows down at high dosages. Additionally, the initial consistency of the OWC paste with DRA-1L remains below 20 Bc and the thickening curve is normal without any abnormal phenomena. Therefore, DRA-1L does not adversely affect the thickening performance of OWC paste at low temperatures [10].

#### 3.3.3. Transition Time of Static Gel Strength of OWC Paste

The transition time of static gel strength is a crucial parameter for evaluating the anti-channeling performance of OWC paste. This transition time represents the time interval required for the static gel strength of OWC paste to increase from 48 Pa to 240 Pa [27]. A short transition time indicates a reduced gas migration distance within the OWC paste matrix, as well as a lower likelihood of dangerous and destructive gas channeling, suggesting that the OWC paste possesses excellent anti-channeling capabilities. It is reported that the relationship between static gelation transition time (Ts) and anti-channeling ability is as follows: Ts ≤ 40 min indicates strong anti-channeling ability; 40 min < Ts < 80 min indicates good anti-channeling ability; Ts > 80 min indicates poor anti-channeling ability; Ts ≥ 110 min indicates extremely poor anti-channeling ability.

**Figure 8:**
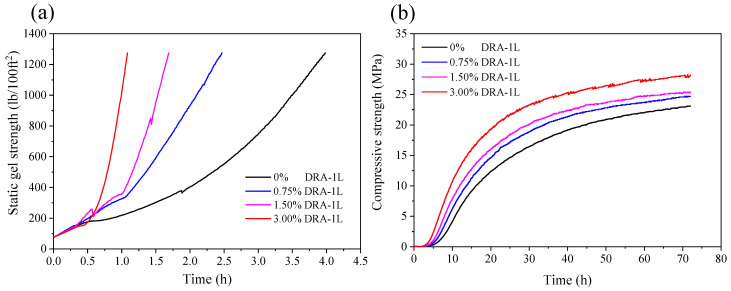
The effect of DRA-1L on the static gel strength (**a**) and compressive strength (**b**) of the OWC paste.

A 5265 static gel strength analyzer was employed to monitor the development of static gel strength and compressive strength of the OWC paste in real time; the results are presented in Figure 8. As the dosage of DRA-1L increased, the static gel strength transition time of the OWC paste decreased, meaning the paste transitioned more quickly from a flow state to a solid state. This reduces the risk of active fluid channeling during the waiting time of the OWC paste. Additionally, the static gel strength transition times for the OWC paste without DRA-1L and the OWC paste with 0.75%, 1.5%, and 3.0% DRA-1L were 134 min, 75 min, 63 min, and 40 min, respectively. These results suggests that DRA-1L can significantly enhance the anti-channeling ability of OWC paste.

#### 3.3.4. Mechanical Strength Development of Cement Stone

**Figure 9:**
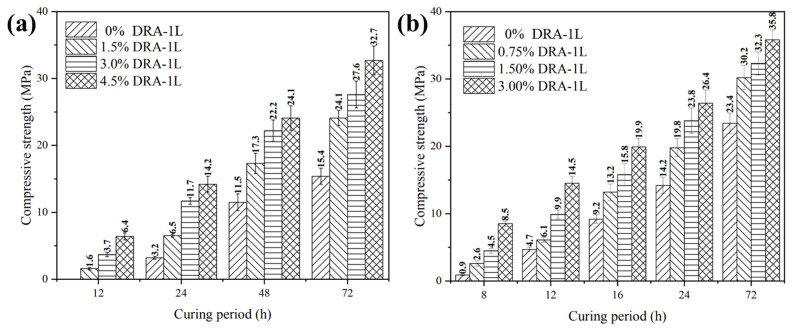
Compressive strength of cement stone with DRA-1L cured at 15 °C (**a**) and 30 °C (**b**).

The ultrasonic method was used to evaluate the development of the compressive strength of cement stone with varying dosages of DRA-1L, with results displayed in Figure 8b. In addition, the effect of DRA-1L on the compressive strength of OWC paste at 15 °C and 30 °C was investigated and the results are presented in Figure 9 and Table 5.

Figure 8b and Figure 9 demonstrate that the addition of DRA-1L significantly enhances the compressive strength of cement stone and accelerates the development of early strength. Notably, this trend becomes more pronounced as the amount of DRA-1L increases within the same curing period. This is because DRA-1L provides ample nucleation sites for the formation of C-S-H gel, thereby reducing the formation barrier and promoting its growth [27]. Additionally, the formation of C-S-H gel consumes Ca^2+^ and SiO_4_^2−^ ions in the liquid phase of the OWC paste, stimulating further dissolution of the mineral phase of Portland cement. DRA-1L continuously promotes cement hydration, leading to the rapid setting of OWC paste and the enhanced early strength of cement stone. Therefore, DRA-1L facilitates the development of the mechanical strength of cement stone.

As depicted in Figure 9 and Table 5, DRA-1L can significantly enhance the compressive strength of cement stone in both early and later curing stages, with strength values increasing as the dosage of DRA-1L rises. At 15 °C and normal pressure, cement stone without DRA-1L had no strength after curing for 12 h, while cement stone with 3.0% DRA-1L achieved a compressive strength exceeding 3.5 MPa, meeting the mechanical requirements for supporting casing and continuing drilling. Compared to cement stone without DRA-1L, the compressive strength of cement stone with 1.5%, 3%, and 4.5% DRA-1L increased by 105%, 270%, and 349% after curing for 24 h, and the compressive strength increased by 57%, 79.8%, and 113% after curing for 72 h, respectively. At 30 °C and normal pressure, the compressive strength of cement stone with 0.75%, 1.5%, and 3% DRA-1L was 2.9 times, 5 times, and 4.5 times that of the cement stone without DRA-1L after curing for 8 h, and the compressive strength increased by 40.3%, 68.5%, and 86.6% after curing for 24 h, respectively. Similarly, the compressive strength increased by 29.1%, 38.5%, and 53.5% after curing for 72 h, respectively.

In summary, DRA-1L can be used across a wide temperature range, which is beneficial to improving the early compressive strength of cement stone at low temperatures. This meets the requirements for low-temperature cementing, significantly shortens the construction period of low-temperature oil and gas wells, and saves drilling costs.

### 3.4. Action Mechanism Analysis of DRA-1L

#### 3.4.1. Cement Hydration Kinetics Analysis

**Figure 10:**
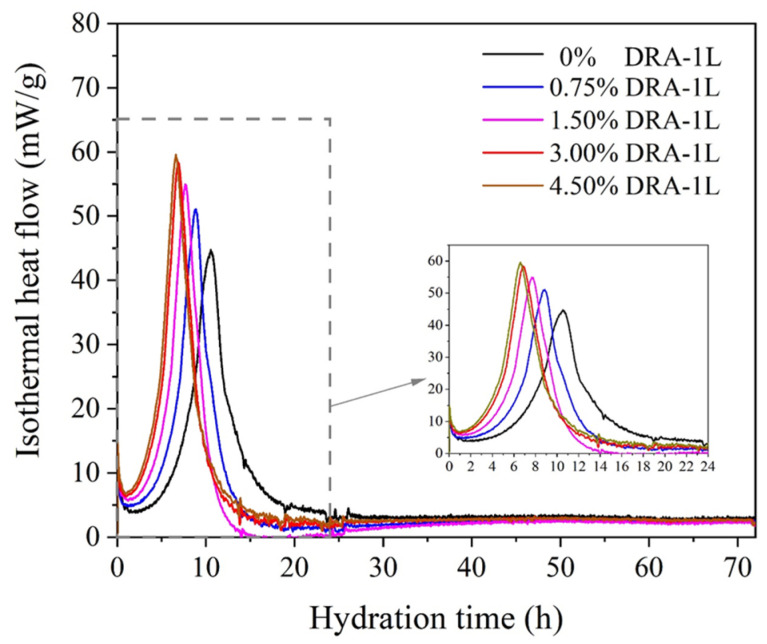
The hydration heat release rate cure of OWC paste with different dosages of DRA-1L.

Isothermal calorimetry was employed to study the effects of DRA-1L on the heat release process during cement hydration. The cement hydration heat release rate curves for OWC pastes with varying dosages of DRA-1L are depicted in Figure 10. As illustrated in Figure 10, increasing the dosage of DRA-1L significantly shortens the induction period of cement hydration, increases the maximum heat release rate, and decreases the time required to reach the maximum heat release rate. Meanwhile, the slope of the cement hydration acceleration period becomes steeper, indicating that DRA-1L can substantially promote cement hydration, leading to the formation of more C-S-H gel and calcium hydroxide (CH) [28]. These above results suggest that nanoscale DRA-1L exhibits a nucleation effect, accelerating the heat release process of cement hydration as well as the generation and growth of hydration products. This enhancement is advantageous for increasing the density of cement stone and improving its early mechanical properties.

#### 3.4.2. Cement Hydration Products Analysis

**Figure 11:**
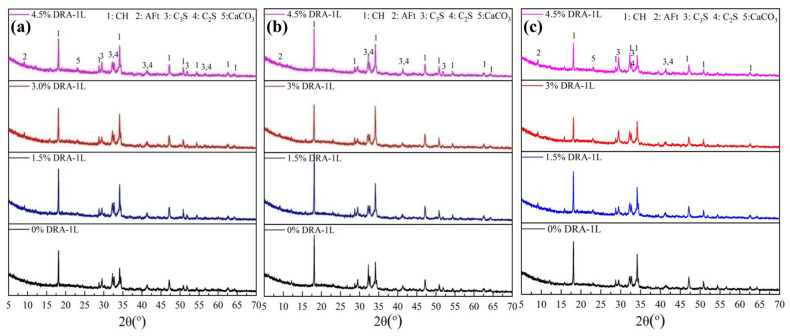
XRD patterns of OWC paste with different dosages of DRA-1L cured for 24 h (**a**), 48 h (**b**), and 72 h (**c**).

XRD measurements were used to qualitatively analyze the effects of DRA-1L on the hydration process of cement minerals, and the results are presented in Figure 11. It is evident that DRA-1L significantly influences the content of mineral components and cement hydration products. As the amount of DRA-1L increases, the peak intensity of CH initially rises and then declines, while the peak intensities of C_2_S and C_3_S gradually decrease. This occurs because DRA-1L accelerates cement hydration and the dissolution of C_2_S and C_3_S, leading to an increase in CH content. Analysis of the heat release process during cement hydration indicates that the hydration reaction stabilizes after 24 h. Consequently, a portion of CH is converted to C-S-H, causing the CH peak intensity to ultimately decrease. The CH content in cement with a constant DRA-1L dosage also follows an initial increase and subsequent decrease as the curing age extends. This is attributed to the continuous dissolution and hydration of C_3_S and C_2_S, which generate CH in the early stages, followed by a transformation into C-S-H and hard wollastonite at later stages. Thus, the addition of DRA-1L provides nucleation sites that serve as C-S-H seed crystals, simultaneously promoting silicate oligomerization and the subsequent growth of hydration products during accelerated cement hydration [29]. Therefore, DRA-1L plays a crucial role in developing and enhancing the mechanical strength of cement stone.

#### 3.4.3. Degree of Cement Hydration Analysis

**Figure 12:**
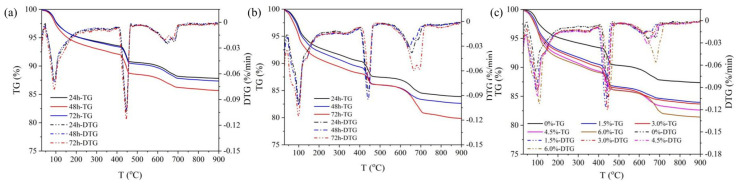
TG-DTG curves of cement stone: (**a**) 0% DRA-1L, (**b**) 4.5% DRA-1L, (**c**) different dosage of DRA-1L cured for 48 h.

Thermogravimetric analysis (TG-DTG) was employed to determine the CH content in cement hydration products and to study the effects of DRA-1L on the degree of cement hydration. As shown in Figure 12, The TG-DTG curves for the cement stone without DRA-1L and with DRA-1L exhibit three stages. The mass loss in the range of 30~350 °C is attributed to the dehydration of C-S-H gel, monosulfur calcium sulfoaluminate (AFm), and ettringite (AFt). The mass loss in the range of 400~550 °C is due to the dehydration of CH. Above 600 °C, the mass loss is primarily caused by the decomposition of calcite [30,31,32,33]. Consequently, the mass loss from 400 °C to 550 °C can be utilized to calculate the relative content of CH and the hydration degree of cement minerals.

As depicted in Figure 12a, the mass loss in the range of 400~550 °C of cement stone without DRA-1L, cured for 24 h, 48 h, and 72 h, was 16.75%, 17.44%, and 19.37%, respectively. This indicates that the CH content gradually increased with the extension of curing age and the hydration reaction of cement minerals became more complete. Figure 12b reveals that the mass loss in the range of 400~550 °C for cement stone with 4.5% DRA-1L, cured for 24 h, 48 h, and 72 h, was 17.94%, 19.14%, and 19.54%, respectively. The evolution trend of CH content in cement stone with 4.5% DRA-1L mirrored that of the OWC paste without DRA-1L. However, the CH content was higher than that of the OWC paste without DRA-1L at the same curing age and it increased slightly in the later curing period, suggesting that DRA-1L promotes CH formation. To further study the effects of DRA-1L on cement hydration products, thermogravimetric curves of cement stones with varying dosages of DRA-1L, cured at 15 °C for 48 h, were measured, with results displayed in Figure 12c. The CH content of cement stone with 0%, 1.5%, 3%, 4.5%, and 6% of DRA-1L was 17.44%, 20.34%, 19.14%, 19.53%, and 20.45%, respectively. The CH content of cement hydration products initially increased and then decreased with increasing DRA-1L dosages, aligning with the XRD analysis results.

In summary, DRA-1L can enhance the dissolution of C_3_S and C_2_S and can expedite the formation and growth of cement hydration products. Its crystal nucleus template effect and large specific surface area facilitate the in situ dispersion and growth of cement hydration products, while also reducing the formation barrier of C-S-H gel.

### 3.5. Application of DRA-1L in Low-Density OWC Paste System

Table 6 presents the comprehensive performance evaluation results for low-density OWC paste containing DRA-1L. The OWC paste system with a density of 1.35 g/cm^3^ (1#) consists of Class G OWC (HSR), 12% ultra-fine cement, 2% micro-silicon, 1.5% fluid loss additive (DRF-1S), 1% dispersant (DRS-1S), 3% DRA-1L, 8% toughened material (DRE-3S), 34% hollow Glass beads, 0.12% retarder (DRH-1L), and 78% water. The OWC paste system with a density of 1.45 g/cm^3^ (2#) is composed of Class G OWC (HSR), 12% ultra-fine cement, 1.5% fluid loss additive (DRF-1S), 3.5% toughened material (DRE-3S), 3% DRA-1L, 23% hollow glass beads, 1% suspension stabilizer (DRY-S1), 1% dispersant (DRS-1S), 0.16% retarder (DRH-1L), and 71% water. The OWC paste system with a density of 1.55 g/cm^3^ (3#) is composed of Class G OWC (HSR), 10% ultra-fine cement, 1.5% fluid loss additive (DRF-1S), 3% toughened material (DRE-3S), 2% DRA-1L, 15% hollow glass beads, 1% suspension stabilizer (DRY-S1), 1% dispersant (DRS-1S), 0.2% retarder (DRH-1L), and 66% water.

As shown in Table 6, the low-temperature and low-density OWC paste with DRA-1L exhibited excellent comprehensive performance, including short ash loading time, good fluidity, low API fluid loss volume (within 50 mL), low initial consistency of OWC paste, and appropriate thickening time, all of which meet the requirements for on-site construction. The strength of cement stone developed rapidly at low temperatures, achieving a compressive strength greater than 10 MPa for 24 h at 30 °C. Consequently, the mechanical performances of cement stone with DRA-1L satisfies the requirements for the mechanical properties of cement sheath and does not adversely affect subsequent drilling operations.

## 4. Conclusions

The C-S-H nanocrystalline nucleus modified with PCE dispersant (DRA-1L) was prepared using a low-temperature solution chemical precipitation method.

(1) The structural characterization of DRA-1L revealed that its crystal structure closely resembles that of amorphous C-S-H gel, with a particle size distribution ranging from 20 to 200 nm.
(2) The performance evaluation of DRA-1L in OWC paste indicated that its addition can enhance rheological properties, significantly reduce the transition time of the static gel strength, and promote the development of cement stone strength and early compressive strength at low temperatures. This effectively addresses the issues of slow strength development and low compressive strength in cement stone at low temperatures.
(3) Additionally, the study of action mechanisms demonstrated that the DRA-1L possesses a crystal nucleus template effect, which can lower the formation barrier of C-S-H gel and accelerate cement hydration. This results in improved compactness and increases the early strength of cement stone.
(4) DRA-1L can be applied to low-temperature OWC paste systems with low density. The low-density OWC paste system with DRA-1L at low temperatures exhibits excellent comprehensive performance, with construction and mechanical properties that meet cementing requirements.

Accordingly, DRA-1L holds significant potential for enhancing the early strength of OWC paste at low temperatures. However, further research is needed on the industrial preparation process of DRA-1L to meet the demands of large-scale on-site applications.

## Figures and Tables

**Table 1:** Chemical and mineral compositions of OWC.

Chemical Component Content (wt%)									Mineral Component Content (wt%)					
Al_2_O_3_	SiO_2_	CaO	MgO	Fe_2_O_3_	SO_3_	K_2_O	Na_2_O	L.O.I ^a^	C_3_A	C_2_S	C_3_S	Gypsum	C_4_AF	f-CaO
3.12	21.89	63.57	1.59	3.47	2.86	0.59	0.42	1.92	6.34	21.17	53.29	3.12	8.97	0.76

^a^ L.O.I refers as the loss on ignition.

**Table 2:** Effect of the Ca/Si molar ratio of the nanocrystalline nucleus on the compressive strength of cement stone.

No.	*n* (Ca(NO_3_)_2_):*n* (Na_2_SiO_3_)	Compressive Strength/MPa			
		4 h	Increase Rate in 4 h/%	8 h	Increase Rate in 8 h/%
1#	—	6.0	0	15.9	0
2#	1:1	11.2	86.7	20.0	25.8
3#	1.25:1	12.3	105.0	22.1	39.0
4#	1.5:1	13.7	128.3	23.8	49.7
5#	1.75:1	18.0	200.0	29.1	83.0
6#	2:1	14.6	143.3	24.5	54.1

**Table 3:** Chemical components of DRA-1L measured by XRF spectrometer.

Chemical Components	CaO	SiO_2_	P_2_O_5_	MgO	SO_3_	Cl	Al_2_O_3_
Content, %	57.59	40.70	0.73	0.24	0.08	0.26	0.10

**Table 4:** Effect of DRA-1L on the rheological properties of OWC paste.

Dosage (%bwoc)	Liquidity (cm)	Six-Speed Reading (Φ3/Φ6/Φ100/Φ300/Φ600)	*n* (Dimensionless)	K (mPa·s*^n^*)
0	20.4 ± 0.4	15/25/52/65/79/121	0.380	3.775
0.75	24.3 ± 0.3	10/21/38/51/72/123	0.581	0.984
1.50	24.7 ± 0.1	6/14/36/48/69/119	0.591	0.884
3.00	25.3 ± 0.3	5/12/34/45/66/126	0.603	0.787
4.50	26.5 ± 0.6	8/18/37/49/74/142	0.630	0.745

Note: The OWC paste with a water–cement ratio of 0.44: OWC, DRA-1L, water.

**Table 5:** Effect of DRA-1L on the compressive strength growth rate of cement stone cured at 15/30 °C.

Cured Conditions	DRA-1L Dosage (wt% bwoc)	Intensity Growth Rate					
		8 h	12 h	16 h	24 h	48 h	72 h
15 °C	1.5	—	—	—	105%	49%	57%
	3.0	—	—	—	270%	94%	80%
	4.5	—	—	—	349%	110%	113%
30 °C	0.75	190%	30%	45%	40%	—	29%
	1.5	400%	111%	73%	69%	—	39%
	3.0	844%	209%	118%	87%	—	54%

**Table 6:** Comprehensive performance of low-density OWC paste with DRA-1L.

No.	ρ (g/cm^3^)	T (s)	Liquidity (cm)	FL_API_ (mL)	Cured Conditions	C_0_ (Bc)	TT (min)	P_24h_ (MPa)		P_48h_ (MPa)	
								30 °C	60 °C	30 °C	60 °C
1#	1.35	36	23.5	34	60 °C × 43 MPa	24	166	10.3	14.2	15.6	24.8
2#	1.45	22	24.2	48	65 °C × 35 MPa	20	251	11.0	17.1	15.7	27.2
3#	1.55	20	24.5	50	65 °C × 35 MPa	22	229	12.3	16.8	17.8	29.2

Note: T is the ash loading time, C_0_ is the initial consistency, TT is the thickening time of OWC paste, and cement stone for compressive strength measurement is cured at normal pressure.

## Data Availability

Data will be made available on request (due to privacy).
